# Changes of Regulatory T Cells and of Proinflammatory and Immunosuppressive Cytokines in Patients with Type 2 Diabetes Mellitus: A Systematic Review and Meta-Analysis

**DOI:** 10.1155/2016/3694957

**Published:** 2016-09-29

**Authors:** Yong-chao Qiao, Jian Shen, Lan He, Xue-zhi Hong, Fang Tian, Yan-hong Pan, Ling Liang, Xiao-xi Zhang, Hai-lu Zhao

**Affiliations:** ^1^Diabetic Systems Medicine, Guangxi Key Laboratory of Excellence, Guilin Medical University, Guilin 541004, China; ^2^Department of Immunology, School of Basic Medical Sciences, Central South University, Changsha, Hunan 410078, China; ^3^Department of Microbiology, The Chinese University of Hong Kong, Shatin, Hong Kong; ^4^Department of Immunology, Faculty of Basic Medicine, Guilin Medical University, Guilin 541004, China

## Abstract

*Objective*. The aim of this study was to investigate the changes of regulatory T cells (Treg), interleukin-6 (IL-6), IL-10, transforming growth factor-*β* (TGF-*β*), and tumor necrosis factor-alpha (TNF-*α*) in patients with type 2 diabetes mellitus (T2DM).* Methods*. We performed a comprehensive search up to July 2016 for all clinical studies about the changes of Treg, IL-6, IL-10, IL-17, TGF-*β*, and TNF-*α* in T2DM patients versus healthy controls.* Results*. A total of 91 articles (5642 cases and 7378 controls) were included for this meta-analysis. Compared with the controls (all *p* < 0.001), the patients had increased serum levels of IL-6, TGF-*β*, and TNF-*α* but decreased the percentage of peripheral CD4^+^CD25^+^Foxp3^+^Treg and serum IL-10 level. Furthermore, the percentage of peripheral CD4^+^CD25^+^Foxp3^+^Treg (*p* < 0.001) and serum IL-10 level (*p* = 0.033) were significantly lower in the patients with complication and in the patients without complication, respectively. No significant changes about the percentage of CD4^+^CD25^+^Treg (*p* = 0.360) and serum IL-17 level (*p* = 0.459) were found in T2DM patients.* Conclusions*. T2DM patients have decreased the percentage of peripheral CD4^+^CD25^+^Foxp3^+^Treg and levels of serum IL-10 but elevated serum levels of IL-6, TGF-*β*, and TNF-*α*. Presence of diabetic complications further lowers the peripheral CD4^+^CD25^+^Foxp3^+^Treg number.

## 1. Introduction

Type 2 diabetes mellitus (T2DM) is one of the most common noncommunicable diseases characterized by insulin resistance and impaired insulin secretion [1, 2]. Metabolic proinflammatory disorder including chronic hyperglycemia and increased levels of circulating cytokines suggests immunological disturbances [3–7], which seriously affects the quality of life of the patients and imposes a large economic burden on the national health care system [8]. Genetic and environmental factors are blamed for T2DM and up to 25% of first-degree relatives of T2DM patients may develop this disease [9]. The origin and development of T2DM were involved in multiple risk factors [10]. Regulatory T cells (Treg) and cytokines play important roles in the development of T2DM.

Treg is a subset of CD4^+^ T cells that maintain peripheral tolerance and suppress antigen specific immune responses by secreting transforming growth factor-*β* (TGF-*β*), interleukin-10 (IL-10), and IL-4 to inhibit autoimmunity [11]. It was found that the ratios of CD4^+^CD25^hi^Treg/Th17 cells and CD4^+^CD25^hi^Treg/Th1 cells were significantly decreased in T2DM patients [12]. Expression of Foxp3, a key player for the development and function of Treg, correlates well with regulatory activity and number of Treg. Indeed, Foxp3 is exclusively expressed in CD4^+^CD25^+^Treg [13–16]. A positive correlation between CD4^+^CD25^+^Foxp3^+^Treg and the enhanced expression of IL-6 on CD4^+^ T cells was observed in T2DM patients [17]. IL-10, as a multifunctional cytokine and secretion of Treg, plays a key role in the inflammatory response that is associated with insulin resistant states and T2DM [18]. Increased levels of IL-17 were found to protect against autoimmune mediated T1DM in nonobese diabetic mice [19]. On the other hand, loss of IL-17 has been associated with disease susceptibility in part because it has been suggested that the absence of IL-17 results in enhanced production of other proinflammatory cytokines [20]. TGF-*β* is also a multifunctional cytokine circulating as a biologically inactive form in human plasma [21, 22]. The TGF-*β* family includes multifunctional molecules that exert specific effects on cell proliferation, differentiation, migration, development, tissue remodeling, and repair [23]. TNF-*α* inhibits the insulin signaling cascade through regulating several pivotal regulatory proteins, such as the insulin receptor substrate (IRS) and Akt substrate 160 in human skeletal muscle* in vitro* [24] and* in vivo* [25]. It has reported that polymorphism of immune genes such as TNF-*α* [26] and TGF-*β* [27] was associated with the development of T2DM. Intriguingly, increased renal production of TGF-*β* was a distinct feature of diabetes [28–31].

Within the past few years, many clinical studies have been focusing on the association of Treg with proinflammatory and immunosuppressive cytokines in T2DM. Despite intensive research efforts, results of these studies have been inconsistent. Therefore, we performed this meta-analysis synthesizing the data from case-control studies to evaluate changes of Treg, IL-6, IL-10, IL-17, TGF-*β*, and TNF-*α* in T2DM patients.

## 2. Materials and Methods

### 2.1. Study Identification and Search Strategy

Our study was conducted according to the Preferred Reporting Items for Systematic Reviews and Meta-Analyses (PRISMA) criteria [32]. We identified relevant studies of Treg, IL-6, IL-10, IL-17, TGF-*β*, and TNF-*α* in T2DM patients by systematically searching PubMed, Wanfang database, Chinese-Cqvip, and CNKI databases from February 1, 1991, to July 15, 2016. The search terms used were as follows: (“interleukin-6” or “IL-6”) or (“interleukin-10” or “IL-10”) or (“interleukin-17” or “IL-17”) or (“transforming growth factor beta” or “TGF-*β*”) or (“tumor necrosis factor alpha” or “TNF-*α*”) or (“regulatory T cells” or “Treg” or “CD4^+^CD25^+^ T cell” or “CD4^+^CD25^+^Foxp3^+^ T cell”) and (“type 2 diabetes mellitus” or “type 2 diabetes” or “diabetes mellitus” or “diabetic patients” or “T2DM” or “DM”). In addition, we also conducted an extensive literature search and articles were further identified in reference lists. Data published in either English or Chinese were included.

### 2.2. Inclusion Criteria

We reviewed all relevant articles using the following inclusion criteria: (1) the study should evaluate the relationship of CD4^+^CD25^+^Foxp3^+^Treg, CD4^+^CD25^+^Treg, IL-6, IL-10, IL-17, TGF-*β*, or TNF-*α* with T2DM patients; (2) the design had to be a case-control study; (3) original data were displayed or could be converted to as mean ± SD; and (4) original report showed no duplicated data.

### 2.3. Quality Assessment and Data Extraction

The data were extracted independently by two reviewers (Yong-chao Qiao and Jian Shen) by using predefined data extraction forms and the quality of all eligible studies was evaluated according to the Newcastle-Ottawa Scale (NOS) [33]. The following information was extracted: (1) name of the first author; (2) date of publication; (3) country of the study; (4) study design; (5) sample size of patients and controls; (6) mean age of the sample; and (7) mean ± SD of patients and controls. In case of disagreement, a third investigator (Hai-lu Zhao) was invited to assess such articles and the disagreements were resolved through discussion.

### 2.4. Statistical Analysis

We presented the data (sample size, mean ± SD) to illustrate the changes of Treg, IL-6, IL-10, IL-17, TGF-*β*, and TNF-*α* in T2DM patients versus healthy controls, and Chi-squared *Q* test and *I* ^2^ statistics were used to assess heterogeneity. When *p* < 0.1 or *I* ^2^ > 50%, the heterogeneity was considered significant and a random effect model was used; otherwise, a fixed-effect model was used. Considering the influence of diabetic complications, patients were divided into two groups (T2DM with complication and T2DM without complication) for subgroup analysis. Regression analysis is also an important method for exploring sources of heterogeneity. We performed sensitivity analysis by limiting the studies of NOS score ≥ 7 or excluding studies with a high risk of bias. Publication bias was examined graphically by constructing Egger's test and *p* < 0.05 was considered to be representative of statistically significant publication bias. Stata 12.0 software was performed in this meta-analysis.

## 3. Results

### 3.1. The Process and Results of Selection

**Figure 1:**
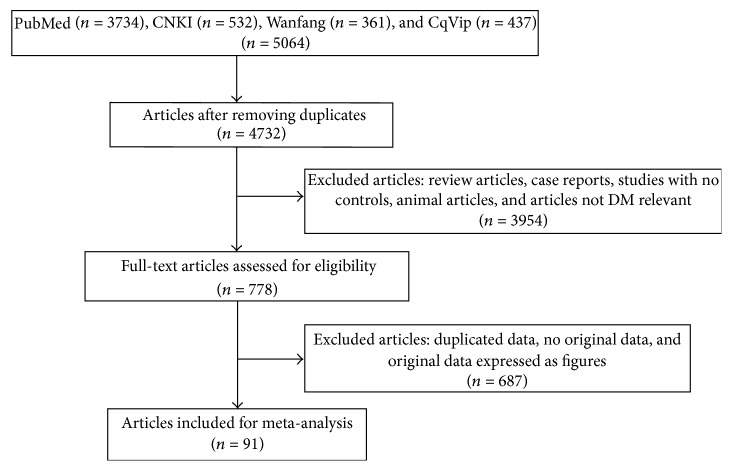
A flow chart of the article search and inclusion process.

The flow chart of the article search and inclusion process was displayed in Figure 1. Based on the search strategy, a total of 5,064 articles were collected and 332 were removed after our initial screening. Furthermore, 3,954 articles were excluded because they were not DM relevant, have no controls, or were animal studies or review articles. Then, we excluded 687 studies because of duplicated data, no original data, or original data expressed with figures. Eventually, this meta-analysis included 91 articles involving 138 case-control studies of 5642 T2DM patients and 7378 healthy controls: 13 for IL-6 [34–46], 22 for TGF-*β* [23, 47–67], 7 for TNF-*α* [34–36, 38, 45, 68, 69], 6 for CD4^+^CD25^+^Foxp3^+^Treg [70–75], 15 for IL-10 [76–90], 18 for CD4^+^CD25^+^Treg [70, 72, 74, 75, 91–104], and 10 for IL-17 [105–114]. Main characteristics of the 91 included studies were listed in Tables 1 [Table tab2] [Table tab3] [Table tab4] [Table tab5] [Table tab6]–7. The case-control study of T2DM with complication was labelled with “*∗*”. NOS results showed high methodological quality.

### 3.2. Results of Meta-Analysis

**Figure 2:**
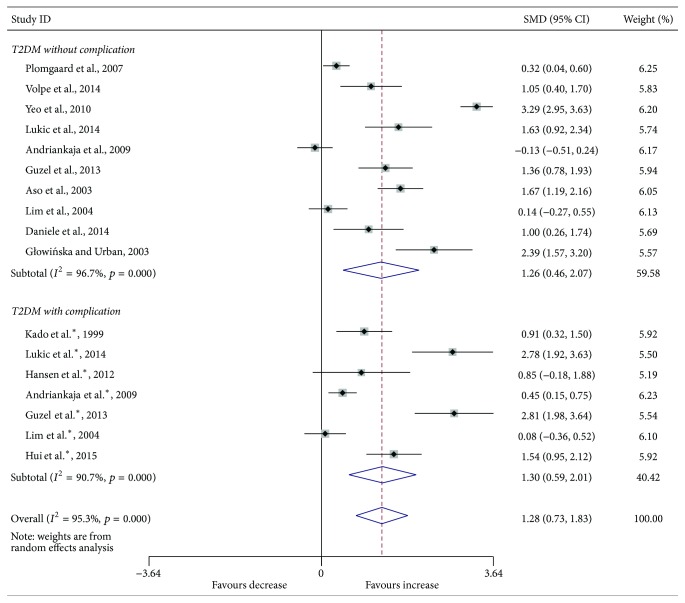
Forest plots for serum IL-6 in T2DM patients and controls with random effects model (T2DM without complication, *p* = 0.002; T2DM with complication, *p* < 0.001; overall, *p* < 0.001). ^*∗*^T2DM with complication.

**Figure 3 fig3:**
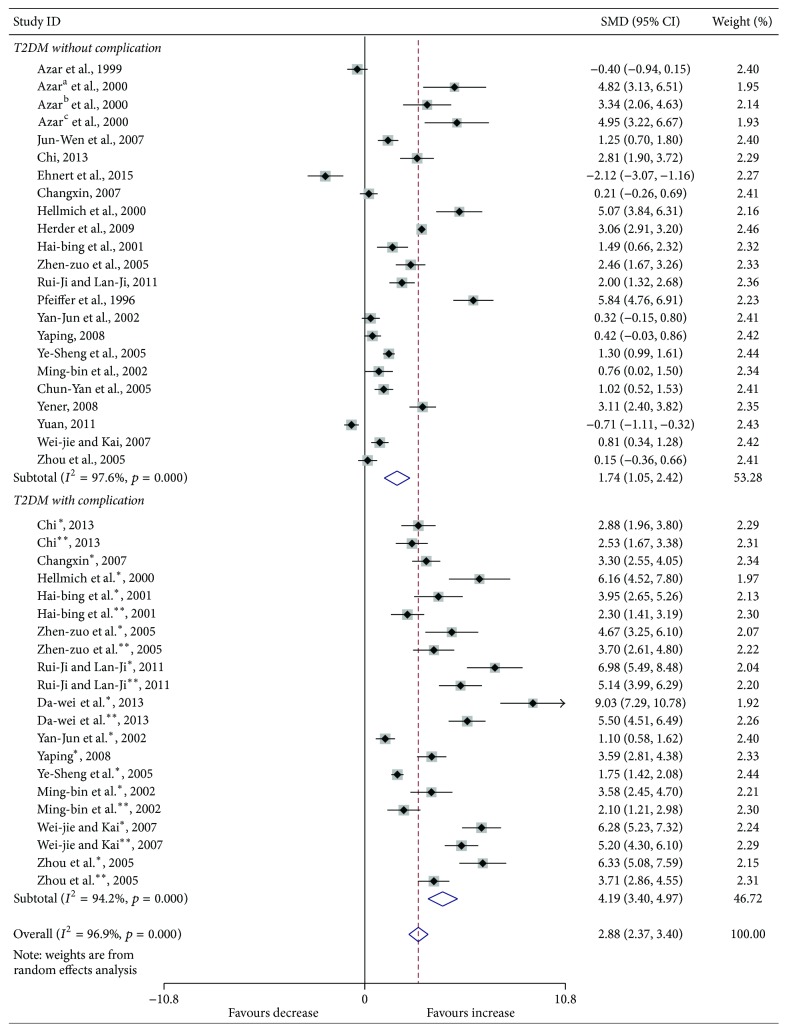
Forest plots for serum TGF-*β* in T2DM patients and controls with random effects model (T2DM without complication, *p* < 0.001; T2DM with complication, *p* < 0.001; overall, *p* < 0.001). ^*∗*^T2DM with complication. ^*∗∗*^T2DM with different complication.

**Figure 4 fig4:**
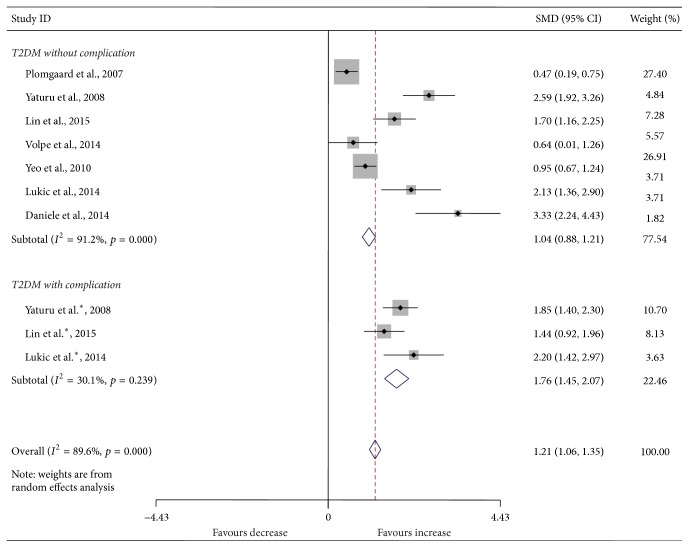
Forest plots for serum TNF-*α* in T2DM patients and controls with random effects model (T2DM without complication, *p* < 0.001; T2DM with complication, *p* < 0.001; overall, *p* < 0.001). ^*∗*^T2DM with complication.

**Figure 5 fig5:**
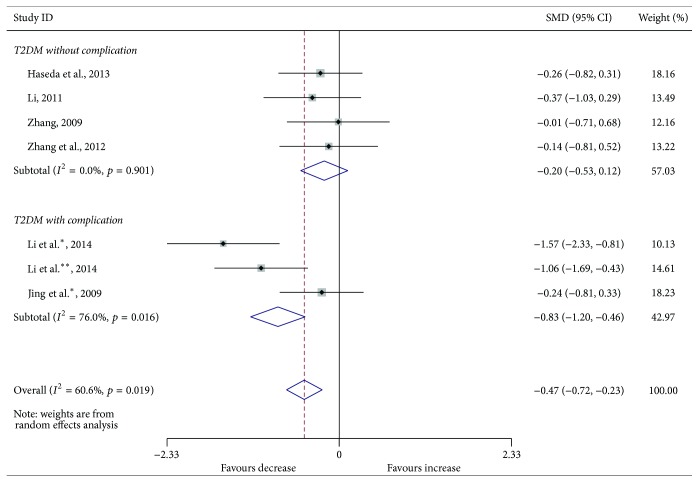
Forest plots for the frequency of CD4^+^CD25^+^Foxp3^+^Treg in T2DM patients and controls with random effects model (T2DM without complication, *p* = 0.211; T2DM with complication, *p* < 0.001; overall, *p* < 0.001). ^*∗*^T2DM with complication. ^*∗∗*^T2DM with different complication.

**Figure 6 fig6:**
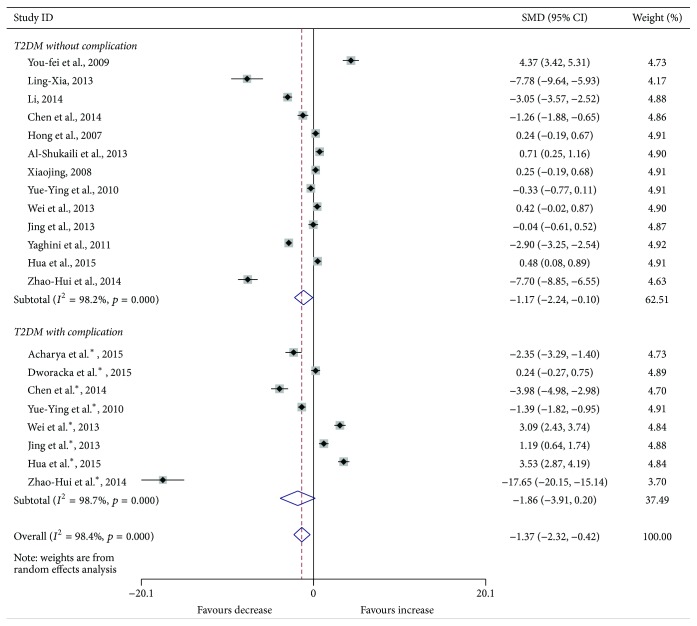
Forest plots for serum IL-10 in T2DM patients and controls with random effects model (T2DM without complication, *p* = 0.033; T2DM with complication, *p* = 0.076; overall, *p* = 0.005). ^*∗*^T2DM with complication.

**Figure 7 fig7:**
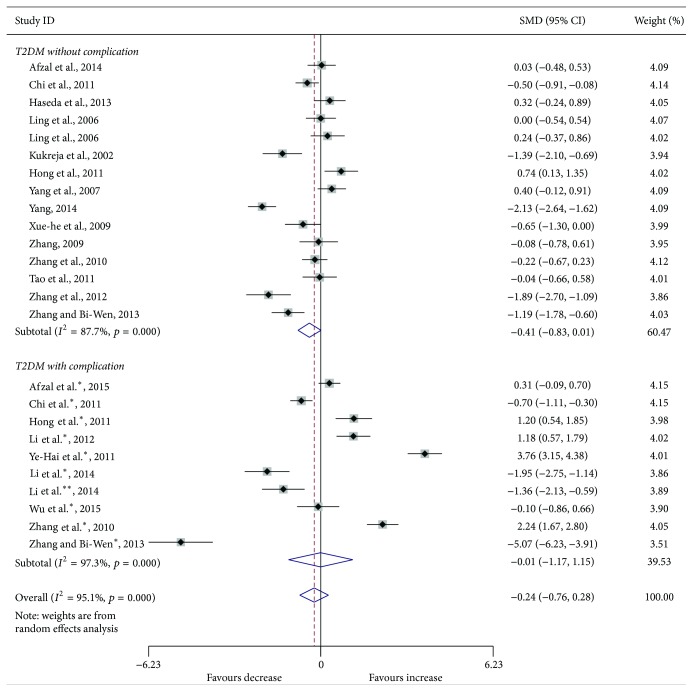
Forest plots for the frequency of CD4^+^CD25^+^Treg in T2DM patients and controls with random effects model (T2DM without complication, *p* = 0.053; T2DM with complication, *p* = 0.987; overall, *p* = 0.360). ^*∗*^T2DM with complication. ^*∗∗*^T2DM with different complication.

**Figure 8:**
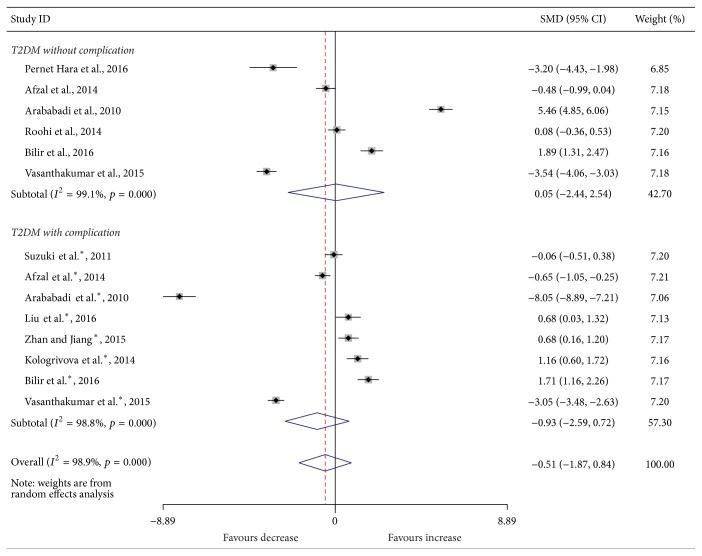
Forest plots for serum IL-17 in T2DM patients and controls with random effects model (T2DM without complication, *p* = 0.969; T2DM with complication, *p* = 0.269; overall, *p* = 0.459). ^*∗*^T2DM with complication.

Compared with the controls, T2DM patients had significantly increased levels of serum IL-6 (SMD, 1.28; 95% CI, 0.73 to 1.83; *p* < 0.001) (Figure 2), TGF-*β* (SMD, 2.88; 95% CI, 2.37 to 3.40; *p* < 0.001) (Figure 3), and TNF-*α* (SMD, 1.56; 95% CI, 1.10 to 2.02; *p* < 0.001) (Figure 4) but significantly decreased the percentage of CD4^+^CD25^+^Foxp3^+^Treg (SMD, −0.47; 95% CI, −0.72 to −0.23; *p* < 0.001) (Figure 5) and the level of serum IL-10 (SMD, −1.37; 95% CI, −2.32 to −0.42; *p* = 0.005) (Figure 6). Changes in the percentage of CD4^+^CD25^+^Treg (SMD, −0.24; 95% CI, −0.76 to 0.28; *p* = 0.360) (Figure 7) and IL-17 (SMD, −0.51; 95% CI, −1.87 to 0.84; *p* = 0.459) (Figure 8) were not significant. Some but not all the results of the meta-analysis displayed significant heterogeneity.

### 3.3. Subgroup Analysis and Regression Analysis

Subgroup analysis was performed to explore the impact of diabetic complication on the changes in Treg and cytokines. As shown in Figures 2 [Fig fig3] [Fig fig4] [Fig fig5] [Fig fig6] [Fig fig7]–8, both T2DM patients with complication and the patients without complication had significantly increased levels of serum IL-6 (Figure 2), TGF-*β* (Figure 3), and TNF-*α* (Figure 4), while not significant changes were found in the percentage of peripheral CD4^+^CD25^+^Treg (Figure 7) and IL-17 (Figure 8). Intriguingly, T2DM patients with complication showed lower percentage of peripheral CD4^+^CD25^+^Foxp3^+^Treg (*p* < 0.001) (Figure 5), whereas patients without complication had decreased levels of serum IL-10 (*p* = 0.033) (Figure 6).

The high heterogeneity existed in some subgroup analysis. In order to explore the source of heterogeneity, we further conducted regression analysis according to the complication as covariate. The results were as follows: TGF-*β* (*t* = 4.08; *p* < 0.001; 95% CI, 1.23 to 3.65), IL-6 (*t* = 0.09; *p* = 0.929; 95% CI, −1.09 to 1.18), TNF-*α* (*t* = 0.34; *p* = 0.740; 95% CI, −1.23 to 1.67), CD4^+^CD25^+^Foxp3^+^Treg (*t* = −2.04; *p* = 0.097; 95% CI, −1.55 to 0.18), IL-10 (*t* = −0.36; *p* = 0.723; 95% CI, −5.33 to 3.77), CD4^+^CD25^+^Treg (*t* = 0.63; *p* = 0.534; 95% CI, −0.96 to 1.81), and IL-17 (*t* = −0.56; *p* = 0.586; 95% CI, −4.84 to 2.86). Therefore, diabetic complication was a key influencing factor for the high heterogeneity in the meta-analysis of TGF-*β* but not the others.

### 3.4. Sensitivity Analysis

Sensitivity analysis was used to assess the stability of the results by excluding studies with high risk of bias and no significant changes in the results were found. We further conducted sensitivity analysis by including studies with high NOS score (≥7) and found that all the results remained consistent.

### 3.5. Publication Bias

**Figure 9:**
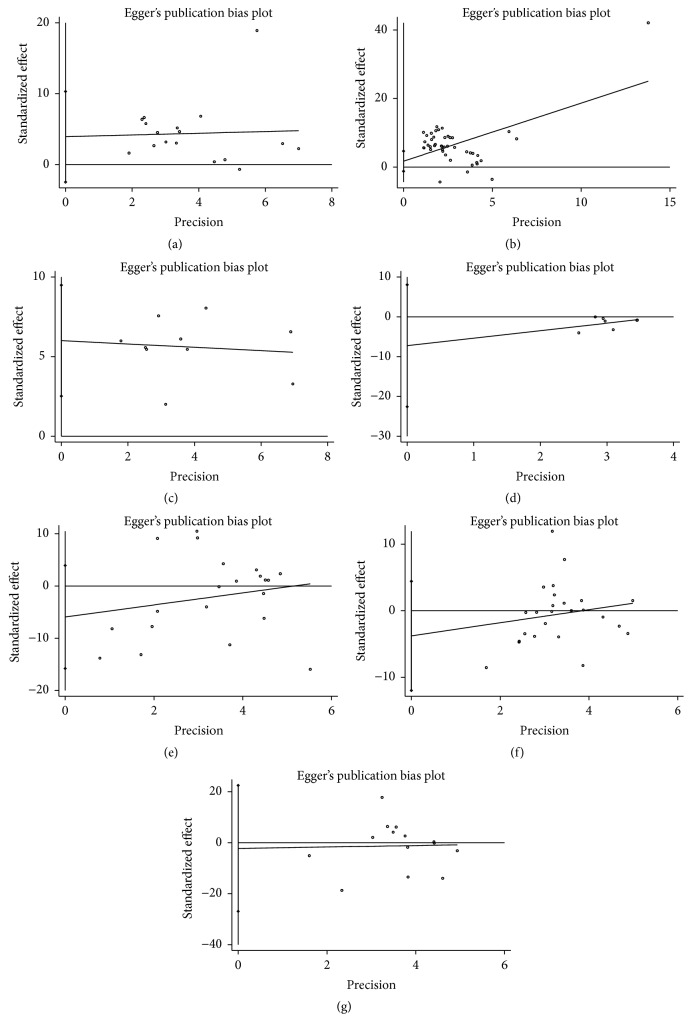
Egger's test about the Treg and cytokines for publication bias ((a) IL-6,* t* = 1.31,* p* = 0.209, CI, −2.46 to 10.33; (b) TGF-*β*,* t* = 1.20,* p* = 0.238, CI, −1.20 to 4.69; (c) TNF-*α*,* t* = 3.98,* p* = 0.004, CI, 2.52 to 9.49; (d) CD4^+^CD25^+^Foxp3^+^Treg,* t* = −1.22,* p* = 0.277, CI, −22.58 to 8.05; (e) IL-10,* t* = −1.26,* p* = 0.223, CI, −15.80 to 3.93; (f) CD4^+^CD25^+^Treg,* t* = −0.95,* p* = 0.351, CI, −11.97 to 4.43; (g) IL-17,* t* = −0.20,* p* = 0.845, CI, −26.99 to 22.44).

Egger's test showed significant publication bias in the meta-analysis of TNF-*α* but not the others (Figure 9).

## 4. Discussion

In this study, we found that the patients with T2DM had increased serum levels of IL-6, TGF-*β*, and TNF-*α* but decreased percentage of peripheral CD4^+^CD25^+^Foxp3^+^Treg and serum IL-10 level. Furthermore, the percentage of peripheral CD4^+^CD25^+^Foxp3^+^Treg and serum IL-10 level were influenced by diabetic complication.

The expression of inflammatory and proinflammatory cytokines from peripheral blood T lymphocyte plays an important role in the development of diabetes and diabetic complications [17]. Many studies have proved the maintenance of immunological self-tolerance by CD4^+^CD25^+^Treg and CD4^+^CD25^+^Foxp3^+^Treg [115]. Treg could suppress inflammatory response through contact inhibition [116]. In this study, the finding of decreased percentage of peripheral CD4^+^CD25^+^Foxp3^+^Treg in T2DM patients indicates that Foxp3 might be a key player for the development and function of Treg. CD4^+^CD25^+^Foxp3^+^Treg differs from CD4^+^CD25^+^Treg. CD4^+^CD25^+^Treg might not sufficiently represent the negative regulatory Treg. Some researchers also considered that the differentiation and function maintenance of Treg were dependent on the expression of the Foxp3, and, consequently, Foxp3 is considered as the key transcriptional factor in Treg cells [117–119].

IL-10 and TGF-*β* secreted by Treg [116, 120] are the biomarkers in T2DM patients [2, 116]. Previous studies suggested that IL-10 could suppress the proliferation of T leukomonocyte and the secretion of cytokines [121], whereas TGF-*β* may sustain the expression of Foxp3 in CD4^+^CD25^+^Treg to enhance immunosuppressive function [122, 123]. Consistent with our findings, several studies have shown a significantly decreased level of serum IL-10 in T2DM patients [88, 124]. Correlation of T2DM with Treg cells and TGF-*β* is generally negative [116].

IL-6 can be released from macrophages and adipocytes in adipose tissue [125–127]. Adipose tissue also produces TNF-*α* to stimulate IL-6 gene expression [128]. A recent investigation has showed that IL-6 could enhance Treg in mice [129]. In the present meta-analysis of T2DM patients, increased levels of serum IL-6, TGF-*β*, and TNF-*α* coexisted with decreased levels of IL-10 and decreased percentage of CD4^+^CD25^+^Foxp3^+^Treg. This finding highlights that the cytokines and growth factors may originate from multiple sources such as macrophages, T cells, and other tissue cells rather than Treg alone. Furthermore, chronic persistent activation of innate immunity and IL-6 secretion occurring in T2DM might inhibit the development of inducible Treg cells.

Th17 cells could produce IL-17, TNF-*α*, and IL-6 and induce inflammation in the pathogenesis of autoimmune diseases [130]. Th17 cells are a major T cell subset implicated in the pathogenesis of multiple sclerosis, rheumatoid arthritis, and psoriasis [131]. A previous study has revealed that not only Th1/Th2 imbalance but also Th17/Treg imbalance can contribute to the pathogenesis of autoimmune diseases such as T1DM as well as proinflammatory disorders and such as T2DM [2]. T2DM patients have elevated serum levels of IL-6, IL-1*β*, and TGF-*β*, the cytokines known to induce Th17 differentiation [131]. Enhanced production of IL-6 and TNF-*α* and decreased levels of serum IL-10 that occurred in T2DM patients may suppress Treg cells and ratios of Treg to Th17 and Th1 cells [132, 133]. The immunocompromised effects on macrophages and lymphocytes likely drive an inflammatory state to contribute to the occurrence of diabetic complications [12]. Here, in this study, no significant changes of Foxp3^+^Treg cells and serum IL-17 levels were found in T2DM subjects without complication. In contrast, decreased Foxp3^+^Treg cells were evident in T2DM subjects with complication. These findings indicate a close correlation of CD4^+^CD25^+^Foxp3^+^Treg and diabetic complication in T2DM.

There is an intimate relationship of the differentiation of Th17 cells with the relative abundance of peripheral CD4^+^CD25^+^Foxp3^+^Treg cells and the serum levels of IL-6, IL-10, and TGF-*β*. Although changes of serum levels of IL-17 were not significant in this meta-analysis of T2DM patients versus controls, IL-17 may be a clue to the possible involvement of Th17 cells in T2DM pathogenesis. Firstly, a decrease of Treg cells might be accompanied by an increase of Th17 cells. The study by Guan et al. has indicated the existence of a developmental switch between Th1/Th17 cells, on one hand, and Th2/Treg cells, on the other hand [134]. Secondly, in the presence of high serum levels of IL-6 and TGF-*β*, as we reported here, differentiation of Th17 cells might be favoured. Lastly, Th17 cells might be, together with innate cells, a primary source of the increased IL-6 levels and might be actively orchestrating the immunity-driven, chronic inflammation of target tissues and organs in T2DM. In this systematic review, the studies examining the number of Th17 cells in T2DM were too scarce for being included in the meta-analysis. Future studies are required to focus on the role of Th17/Treg and products of the Th17 cells in the pathogenesis of T2DM and associated complications.

Diabetic complications such as retinopathy, nephropathy, and cardiovascular disease affect immune cells and cytokines in type 2 diabetes [135, 136]. Actually, urinary TGF-*β* levels are elevated in the presence of microalbuminuria and overt proteinuria [137]. Additionally, elevated plasma TGF-*β* may reflect the state of hyperglycemia in T2DM patients [48]. Systemic inflammation in T2DM is linked to the development of diabetic complications [138, 139]. Yet, the mechanism of immune alteration in T2DM and diabetic complication remains unclear. In this meta-analysis, diabetic complication indeed has an impact on the percentage of peripheral CD4^+^CD25^+^Foxp3^+^Treg and level of serum IL-10. The percentage of Treg cells and levels of cytokines in T2DM may also depend on ethnicity, sex, weight, age, and disease duration.

Publication bias might influence the interpretation of our final results. The results of Egger's tests explain that no publication bias existed in all comparisons except for TNF-*α*. The publication bias in this meta-analysis might be attributed to studies of small samples and positive results published more easily than negative reports.

There are some limitations in this meta-analysis when interpreting the findings. Firstly, we have selected random effect model to synthesize SMD because of the high heterogeneity existing in some comparisons, but this selection may affect the accuracy of outcome. Secondly, we could not conduct further subgroup analysis of gender, weight, and disease duration because most of the included studies lack sufficient original data. Thirdly, articles published in Chinese or English are included, while unpublished data and papers published in other languages are unknown.

## 5. Conclusions

In summary, T2DM patients and the patients with diabetic complication have decreased immunosuppressive CD4^+^CD25^+^Foxp3^+^Treg cells and increased proinflammatory IL-10, TGF-*β*, and TNF-*α*. The presence of diabetic complication has an impact on the compromised immunosuppression. Significant interaction exists between immune and metabolic homeostasis.

## Figures and Tables

**Table 1:** Characteristics of studies about IL-6 (pg/mL) included in this meta-analysis.

Author	Year	Country	Case				Control				NOS score
			SZ	M/F	Mean	SD	SZ	M/F	Mean	SD	
Plomgaard^a^ [34]	2007	Denmark	96	72/24	1.63	1.22	103	70/33	1.27	1.04	7
Volpe [35]	2014	Brazil	29	10/19	119.1	23.3	16	5/11	97.6	13.5	8
Yeo [36]	2010	Korea	55	27/28	2.3	0.35	488	257/231	1.8	0.11	8
Kado^*∗*^ [37]	1999	Japan	57	NR	3.48	3.29	15	NR	0.784	0.9	7
Lukic [38]	2014	Serbia	30	NR	11.77	6.09	15	NR	3.48	1.48	8
Lukic^*∗*^ [38]	2014	Serbia	30	NR	15.46	5.15	15	NR	3.48	1.48	8
Hansen^*∗*,a^ [39]	2012	Denmark	8	NR	4.6	5.2	8	NR	1.4	1.15	7
Andriankaja [40]	2009	USA	30	NR	1.5	1.4	310	NR	1.8	2.3	8
Andriankaja^*∗*^ [40]	2009	USA	50	NR	2.9	3.2	310	NR	1.8	2.3	8
Guzel [41]	2013	Turkey	28	NR	3.21	1.24	30	19/14	1.73	0.93	8
Guzel^*∗*^ [41]	2013	Turkey	17	NR	6.67	2.67	30	19/14	1.73	0.93	8
Aso [42]	2003	Japan	42	22/20	3.15	1.53	48	NR	1.29	0.52	7
Lim^a^ [43]	2004	England	56	31/25	1.9	8.02	39	21/18	1	1.59	8
Lim^*∗*,a^ [43]	2004	England	41	30/11	2.6	26.95	39	21/18	1	1.59	7
Hui^*∗*^ [44]	2015	China	48	20/28	16.17	8.36	20	13/7	5.2	2.03	8
Daniele [45]	2014	USA	17	13/4	2.1	0.4	15	7/8	1.7	0.4	8
Głowińska [46]	2003	Polish	28	NR	6.5	1.4	15	NR	2.3	2.3	7

DM: diabetes mellitus; SZ: sample size; M/F: man/female; SD: standard deviation; NR: not reported. ^*∗*^T2DM with complication and ^a^data (mean ± SD) converted from mean (95% CI).

**Table 2 tab2:** Characteristics of studies about TGF-*β* (*μ*g/L) included in this meta-analysis.

Author	Year	Country	Case				Control				NOS score
			SZ	M/F	Mean	SD	SZ	M/F	Mean	SD	
Azar [47]	1999	Lebanon	26	8/18	0.558	0.107	27	10/17	0.593	0.064	8
Azar^a^ [48]	2000	Lebanon	8	3/5	10.8	2.3	15	9/6	4.1	0.5	8
Azar^b^ [48]	2000	Lebanon	9	2/7	9.9	2.8	15	9/6	4.1	0.5	8
Azar^c^ [48]	2000	Lebanon	8	5/3	10.7	2.2	15	9/6	4.1	0.5	8
Jun-Wen [49]	2007	China	61	35/26	315.9	224.59	19	10/9	68.47	31.75	7
Chi [50]	2013	China	20	11/9	18.55	2.67	18	10/8	8.97	4.087	8
Chi^*∗*^ [50]	2013	China	20	9/11	19.04	2.87	18	10/8	8.97	4.087	8
Chi^*∗∗*^ [50]	2013	China	21	13/8	18.12	3.17	18	10/8	8.97	4.087	8
Ehnert [51]	2015	Germany	14	7/7	35.3	2.4	13	7/6	39.5	1.4	8
Changxin [52]	2007	China	34	24/10	36.2	8.8	35	24/11	34.4	8.2	7
Changxin^*∗*^ [52]	2007	China	31	21/10	69.4	12.8	35	24/11	34.4	8.2	7
Hellmich [53]	2000	Germany	35	16/19	9.5	2.1	12	5/7	0.24	0.03	8
Hellmich^*∗*^ [53]	2000	Germany	23	8/15	10.8	2.1	12	5/7	0.24	0.03	8
Herder [54]	2009	Germany	460	255/205	35.8	0.4	1474	724/750	35	0.2	8
Hai-bing [55]	2001	China	14	7/7	35.02	6.7	15	7/8	23.95	8.01	6
Hai-bing^*∗*^ [55]	2001	China	13	5/8	58.58	9.56	15	7/8	23.95	8.01	6
Hai-bing^*∗∗*^ [55]	2001	China	18	9/9	39.31	5.35	15	7/8	23.95	8.01	6
Zhen-zuo [56]	2005	China	27	14/13	41	15.57	18	9/9	10.04	5.33	7
Zhen-zuo^*∗*^ [56]	2005	China	12	7/5	66.35	18.04	18	9/9	10.04	5.33	7
Zhen-zuo^*∗∗*^ [56]	2005	China	18	9/9	53.31	15.64	18	9/9	10.04	5.33	7
Rui-Ji [57]	2011	China	32	NR	35	5	20	NR	25	5	6
Rui-Ji^*∗*^ [57]	2011	China	31	NR	69	7	20	NR	25	5	6
Rui-Ji^*∗∗*^ [57]	2011	China	32	NR	54	6	20	NR	25	5	6
Da-wei^*∗*^ [58]	2013	China	15	NR	234.2	29.8	45	NR	69.4	12.5	7
Da-wei^*∗∗*^ [58]	2013	China	32	NR	155.6	19.3	45	NR	69.4	12.5	7
Pfeiffer [59]	1996	Germany	44	22/22	7.9	1	28	16/12	3.1	0.4	8
Yan-Jun [60]	2002	China	34	16/18	147.03	22.57	35	17/18	136.97	37.96	7
Yan-Jun^*∗*^ [60]	2002	China	31	15/16	170.65	18.74	35	17/18	136.97	37.96	7
Yaping [61]	2008	China	44	NR	35.4	7.1	35	NR	32.5	6.8	7
Yaping^*∗*^ [61]	2008	China	32	NR	68.2	12.5	35	NR	32.5	6.8	7
Ye-Sheng [62]	2005	China	92	42/50	36.89	9.75	105	50/55	25.46	7.88	6
Ye-Sheng^*∗*^ [62]	2005	China	91	40/51	41.57	10.55	105	50/55	25.46	7.88	6
Ming-bin [63]	2002	China	15	NR	25.85	6.09	15	8/7	21.4	5.62	6
Ming-bin^*∗*^ [63]	2002	China	18	NR	38.53	3.98	15	8/7	21.4	5.62	6
Ming-bin^*∗∗*^ [63]	2002	China	16	NR	31.15	3.51	15	8/7	21.4	5.62	6
Chun-Yan [64]	2005	China	92	NR	45.57	21.78	20	NR	24.58	12.61	7
Yener [23]	2008	Turkey	39	18/21	29.84	7.04	30	16/14	11.37	4.06	8
Yuan [65]	2011	China	51	37/14	1.7399	0.4846	55	40/15	2.1045	0.5327	7
Wei-jie [66]	2007	China	36	19/17	23.35	3.7	40	23/17	20.35	3.7	7
Wei-jie^*∗*^ [66]	2007	China	45	25/20	55.28	6.8	40	23/17	20.35	3.7	7
Wei-jie^*∗∗*^ [66]	2007	China	45	23/22	41.31	4.3	40	23/17	20.35	3.7	7
Zhou [67]	2005	China	30	NR	31.12	12.39	30	NR	29.4	10.62	8
Zhou^*∗*^ [67]	2005	China	30	NR	136.6	21.45	30	NR	29.4	10.62	8
Zhou^*∗∗*^ [67]	2005	China	30	NR	79.63	15.96	30	NR	29.4	10.62	8

DM: diabetes mellitus; SZ: sample size; M/F: man/female; SD: standard deviation; NR: not reported. ^*∗*,*∗∗*^T2DM with different complication; ^a,b,c^T2DM with different duration limited.

**Table 3 tab3:** Characteristics of studies about TNF-*α* (pg/mL) included in this meta-analysis.

Author	Year	Country	Case				Control				NOS score
			SZ	M/F	Mean	SD	SZ	M/F	Mean	SD	
Plomgaard^a^ [34]	2007	Denmark	96	72/24	2.72	0.8	103	70/33	2.4	0.54	8
Yaturu^*∗*^ [68]	2008	USA	50	NR	4	0.36	59	NR	3.4	0.29	7
Yaturu [68]	2008	USA	26	NR	4.2	0.47	39	NR	3.2	0.32	8
Lin [69]	2015	China	42	20/22	5.49	1.48	30	14/16	3.46	0.58	8
Lin^*∗*^ [69]	2015	China	45	25/20	6.82	2.97	30	14/16	3.46	0.58	8
Volpe [35]	2014	Brazil	29	10/19	78.7	32.7	16	5/11	58.5	29.5	7
Yeo [36]	2010	Korea	55	27/28	1.5	0.95	488	257/231	1.1	0.31	7
Lukic [38]	2014	Serbia	30	NR	1.53	0.42	15	NR	0.71	0.3	8
Lukic^*∗*^ [38]	2014	Serbia	30	NR	1.54	0.41	15	NR	0.71	0.3	8
Daniele [45]	2014	USA	17	13/4	2.5	0.3	15	7/8	1.5	0.3	7

DM: diabetes mellitus; SZ: sample size; M/F: man/female; SD: standard deviation; NR: not reported. ^*∗*^T2DM with complication and ^a^data (mean ± SD) converted from mean (95% CI).

**Table 4 tab4:** Characteristics of studies about the percentage of CD4^+^CD25^+^Foxp3^+^Tregs (%) in the CD4^+^ lymphocyte included in this meta-analysis.

Author	Year	Country	Case				Control				NOS score
			SZ	M/F	Mean	SD	SZ	M/F	Mean	SD	
Haseda [70]	2013	Japan	20	8/12	4.94	1.78	30	10/20	5.36	1.54	8
Li [71]	2011	China	18	14/4	2.8	8.54	18	12/6	5.01	0.13	7
Li^*∗*^ [72]	2014	China	15	NR	2.32	0.5	21	13/8	4.07	1.39	8
Li^*∗∗*^ [72]	2014	China	23	NR	2.91	0.73	21	13/8	4.07	1.39	8
Jing^*∗*^ [73]	2009	China	60	33/27	3.2733	1.5835	15	8/7	3.6216	0.6938	8
Zhang [74]	2009	China	17	9/8	5.02	3.59	15	8/7	5.07	3.26	7
Zhang [75]	2012	China	16	11/5	2.21	0.92	19	7/12	2.32	0.6	6

DM: diabetes mellitus; SZ: sample size; M/F: man/female; SD: standard deviation; NR: not reported. ^*∗*,*∗∗*^T2DM with different complication.

**Table 5 tab5:** Characteristics of studies about IL-10 (pg/mL) included in this meta-analysis.

Author	Year	Country	Case				Control				NOS score
			SZ	M/F	Mean	SD	SZ	M/F	Mean	SD	
Acharya^*∗*^ [76]	2015	India	15	NR	11.35	0.97	15	NR	15.83	2.52	8
Dworacka^*∗*^ [77]	2015	Poland	30	17/13	4.1	1.5	30	12/13	3.8	0.9	7
You-fei [78]	2009	China	30	14/16	45.859	7.34	30	13/17	18.181	5.145	7
Ling-Xia [79]	2013	China	20	10/10	8.41	1.22	20	10/10	17.56	1.13	7
Li [80]	2014	China	63	4/6	15.69	3.22	57	5/5	24.13	2.17	8
Chen [81]	2014	China	24	NR	5.8	0.9	25	19/11	6.93	0.89	7
Lu^*∗*^ [81]	2014	China	22	NR	3.12	1.03	25	19/11	6.93	0.89	7
Hong [82]	2007	China	46	22/24	4.61	1.2	39	19/20	4.36	0.84	7
Al-Shukaili [83]	2013	Oman	57	28/29	6.95	6	30	20/10	2.9	5.15	8
Xiaojing [84]	2008	China	42	27/15	4.36	2.64	40	25/15	3.64	3.15	7
Yue-Ying [85]	2010	China	34	19/15	4	1.4	50	22/28	4.5	1.6	8
Yue-Ying^*∗*^ [85]	2010	China	50	24/26	2.7	0.9	50	22/28	4.5	1.6	8
Wei [86]	2013	China	39	NR	5.11	1.33	40	NR	4.56	1.27	7
Wei^*∗*^ [86]	2013	China	39	NR	10.52	2.43	40	NR	4.56	1.27	7
Jing [87]	2013	China	20	11/9	5	2.16	30	NR	5.07	1.32	7
Jing^*∗*^ [87]	2013	China	30	19/11	7.58	2.67	30	NR	5.07	1.32	7
Yaghini [88]	2011	Iran	131	NR	9.53	2.27	120	NR	16.11	2.27	7
Hua [89]	2015	China	61	NR	6.98	2.84	40	NR	5.83	1.37	8
Hua^*∗*^ [89]	2015	China	52	NR	30.7	9.28	40	NR	5.83	1.37	8
Zhao-Hui [90]	2014	China	50	26/24	8.39	1.18	50	24/26	17.63	1.22	8
Zhao-Hui^*∗*^ [90]	2014	China	50	28/22	1.86	0.33	50	24/26	17.63	1.22	8

DM: diabetes mellitus; SZ: sample size: M/F: man/female; SD: standard deviation; NR: not reported. ^*∗*^T2DM with complication.

**Table 6 tab6:** Characteristics of studies about the percentage of CD4^+^CD25^+^Tregs (%) in the CD4^+^ lymphocyte included in this meta-analysis.

Author	Year	Country	Case				Control				NOS score
			SZ	M/F	Mean	SD	SZ	M/F	Mean	SD	
Afzal [91]	2014	Pakistan	30	5/25	14.68	6.21	30	21/9	14.53	4.84	8
Afzal^*∗*^ [91]	2014	Pakistan	152	51/101	16.47	6.56	30	21/9	14.53	4.84	8
Chi [92]	2011	China	52	30/22	9.39	2.12	40	20/20	10.43	2.07	7
Chi^*∗*^ [92]	2011	China	68	39/29	8.99	2.03	40	20/20	10.43	2.07	7
Haseda [70]	2013	Japan	20	8/12	27.7	11.1	30	10/20	24.9	6.5	7
Ling [93]	2006	China	25	12/13	1.3	0.6	27	13/14	1.3	0.4	8
Ling [93]	2006	China	20	13/7	13.76	3.27	21	10/11	12.98	3.14	8
Kukreja [94]	2002	USA	15	2/13	6.3	0.48	26	12/14	6.9	0.4	8
Hong [95]	2011	China	20	10/10	5.35	2.12	25	15/10	4.06	1.39	7
Hong^*∗*^ [95]	2011	China	18	10/8	9.8	7.27	25	15/10	4.06	1.39	7
Li^*∗*^ [96]	2012	China	20	10/10	6.24	1.96	30	18/12	4.24	1.5	7
Ye-Hai^*∗*^ [97]	2011	China	81	NR	6.05	1.2	38	NR	2.01	0.73	7
Li^*∗*^ [72]	2014	China	15	NR	9.14	2.21	21	13/8	15.18	3.6	7
Li^*∗∗*^ [72]	2014	China	13	NR	10.69	2.75	21	13/8	15.18	3.6	7
Wu^*∗*^ [98]	2015	China	10	NR	2.1	1.5	20	12/8	2.3	2.2	8
Yang [99]	2007	China	30	18/12	9.84	4.78	30	18/12	8.16	3.65	8
Yang [100]	2014	China	43	19/24	2.02	0.43	52	28/24	2.89	0.39	6
Xue-he [101]	2009	China	22	9/13	6.79	1.75	17	8/9	7.84	1.45	7
Zhang [74]	2009	China	17	9/8	8.5	4.16	15	8/7	8.83	3.87	8
Zhang [102]	2010	China	37	24/13	0.5	0.8	38	21/17	0.7	1	7
Zhang^*∗*^ [102]	2010	China	40	26/14	4.7	2.3	38	21/17	0.7	1	7
Tao [103]	2011	China	20	NR	3.74	0.89	20	NR	3.78	0.95	8
Zhang [75]	2012	China	16	11/5	5.29	2.6	19	7/12	9.75	2.13	7
Zhang [104]	2013	China	36	19/17	8.8	3.6	20	11/9	12.4	1.5	7
Zhang^*∗*^ [104]	2013	China	30	16/14	6.05	1.06	20	11/9	12.4	1.5	7

DM: diabetes mellitus; SZ: sample size; M/F: man/female; SD: standard deviation; NR: not reported. ^*∗*,*∗∗*^T2DM with different complication.

**Table 7:** Characteristics of studies about IL-17 (pg/mL) included in this meta-analysis.

Author	Year	Country	Case				Control				NOS score
			SZ	M/F	Mean	SD	SZ	M/F	Mean	SD	
Pernet Hara [105]	2016	Brazil	15	0/15	6.98	1.11	10	0/10	16.2	4.39	8
Suzuki^*∗*^ [106]	2011	Japan	56	25/31	147.39	113.83	30	2/28	154.52	117.99	7
Afzal [107]	2014	Pakistan	30	5/25	415.01	483.4	30	21/9	718.05	756.55	7
Afzal^*∗*^ [107]	2014	Pakistan	152	51/101	375.95	468.19	30	21/9	718.05	756.55	7
Arababadi [108]	2010	Iran	100	41/59	13.7	2.34	100	40/60	4.43	0.54	8
Arababadi^*∗*^ [108]	2010	Iran	100	38/62	0.94	0.29	100	40/60	4.43	0.54	8
Liu^*∗*^ [109]	2016	China	19	10/9	21.4	5.9	20	8/12	17.3	6.2	7
Roohi [110]	2014	India	38	19/19	6.61	4.97	40	22/18	6.22	4.64	8
Zhan^*∗*^ [111]	2015	China	30	14/16	42.24	67.7	30	14/16	9.33	8.15	8
Kologrivova^*∗*a^ [112]	2014	Russia	35	17/18	100.83	42.7	24	NR	45.29	54.85	7
Bilir^b^ [113]	2016	Turkey	33	15/18	466.1	183.075	33	15/18	205.2	67.725	6
Bilir^*∗*b^ [113]	2016	Turkey	37	17/20	454.9	189.825	33	15/18	205.2	67.725	6
Vasanthakumar^c^ [114]	2015	India	65	38/27	59.8	2.8	88	35/53	94.7	12.75	6
Vasanthakumar^*∗*c^ [114]	2015	India	97	57/40	67.4	2.275	88	35/53	94.7	12.75	6

DM: diabetes mellitus; SZ: sample size; M/F: man/female; SD: standard deviation; NR: not reported. ^*∗*^T2DM with complication; ^a^data converted from median (interquartile); ^b^data converted from median (range); ^c^data converted from geometrical mean (range).

## Abbreviations

T2DM: Type 2 diabetes mellitus
Treg: Regulatory T cells
IL-6: Interleukin-6
IL-10: Interleukin-10
IL-17: Interleukin-17
TGF-*β*: Transforming growth factor-beta
TNF-*α*: Tumor necrosis factor-alpha.
